# Bivalent vaccine platform based on Japanese encephalitis virus (JEV) elicits neutralizing antibodies against JEV and hepatitis C virus

**DOI:** 10.1038/srep28688

**Published:** 2016-06-27

**Authors:** Ryohei Saga, Akira Fujimoto, Noriyuki Watanabe, Mami Matsuda, Makoto Hasegawa, Koichi Watashi, Hideki Aizaki, Noriko Nakamura, Shigeru Tajima, Tomohiko Takasaki, Eiji Konishi, Takanobu Kato, Michinori Kohara, Haruko Takeyama, Takaji Wakita, Ryosuke Suzuki

**Affiliations:** 1 Department of Virology II, National Institute of Infectious Diseases, 1-23-1, Toyama, Shinjuku-ku, Tokyo 162-8640, Japan; 2 Department of Life Science and Medical Bioscience, Waseda University, 2-2, Wakamatsu-cho, Shinjuku-ku, Tokyo 162-8480, Japan; 3 Department of Bioscience, Faculty of Bioscience, Nagahama Institute of Bio-Science and Technology, 1266 Tamura-cho, Nagahama-shi, Shiga 526-0829, Japan; 4 Pharmaceutical Research Laboratories, Toray Industries, Inc., 6-10-1, Tebiro, kanagawa-shi, Kanagawa 248-0036, Japan; 5 Department of Virology I, National Institute of Infectious Diseases, 1-23-1, Toyama, Shinjuku-ku, Tokyo 162-8640, Japan; 6 BIKEN Endowed Department of Dengue Vaccine Development Faculty of Tropical Medicine, Mahidol University, 420/6 Ratchawithi Road, Ratchahewi, Bangkok 10440, Thailand; 7 BIKEN Endowed Department of Dengue Vaccine Development, Research Institute for Microbial Diseases, Osaka University, 3-1 Yamadaoka, Suita, Osaka 565-0871, Japan; 8 Department of Microbiology and Cell Biology, Tokyo Metropolitan Institute of Medical Science, 2-1-6, Kamikitazawa, Setagaya-ku, Tokyo 156-8506, Japan

## Abstract

Directly acting antivirals recently have become available for the treatment of hepatitis C virus (HCV) infection, but there is no prophylactic vaccine for HCV. In the present study, we took advantage of the properties of Japanese encephalitis virus (JEV) to develop antigens for use in a HCV vaccine. Notably, the surface-exposed JEV envelope protein is tolerant of inserted foreign epitopes, permitting display of novel antigens. We identified 3 positions that permitted insertion of the HCV E2 neutralization epitope recognized by HCV1 antibody. JEV subviral particles (SVP) containing HCV-neutralization epitope (SVP-E2) were purified from culture supernatant by gel chromatography. Sera from mice immunized with SVP-E2 inhibited infection by JEV and by trans-complemented HCV particles (HCVtcp) derived from multi-genotypic viruses, whereas sera from mice immunized with synthetic E2 peptides did not show any neutralizing activity. Furthermore, sera from mice immunized with SVP-E2 neutralized HCVtcp with N415K escape mutation in E2. As with the SVP-E2 epitope-displaying particles, JEV SVPs with HCV E1 epitope also elicited neutralizing antibodies against HCV. Thus, this novel platform harboring foreign epitopes on the surface of the particle may facilitate the development of a bivalent vaccine against JEV and other pathogens.

Hepatitis C virus (HCV) is a major cause of chronic liver disease, leading to chronic hepatitis, cirrhosis, and hepatocellular carcinoma. Nearly 150 million people are chronically infected with HCV worldwide^1^. Recently, HCV-specific direct-acting antivirals (DAAs) have been developed that provide increased rates of sustained virological response^2^. However, viruses resistant to DAAs have been observed clinically, and have been associated with treatment failure^3^,^4^. Moreover, the new treatments are expensive, meaning that treatment may not be available to many patients. An effective prophylactic HCV vaccine still remains essential for the control and eradication of this pathogen.

HCV is a positive-strand RNA virus and belongs to the Hepacivirus genus of the family Flaviviridae. HCV has a highly variable genome with multiple genotypes and subgenotypes^5^. In addition, due to the low fidelity of HCV RNA-dependent RNA polymerase, which possesses no proofreading activity, HCV exists as multiple closely related but distinct viral quasispecies even in an infected individual. The high mutation rate of HCV creates wide genetic diversity for the virus, contributing to immune evasion while also representing a major obstacle for the development of a prophylactic HCV vaccine.

Viral clearance has been shown to be associated with a rapid induction of neutralizing antibodies during the early phase of infection in single-source outbreaks of HCV^6^. In addition, neutralizing antibodies were shown to abrogate established HCV infection *in vitro* and in a human liver chimeric mouse model^7^. These data suggest that neutralizing antibodies might play a role in controlling infection. Various antigens, including recombinant glycoprotein E1/E2 with adjuvant^8^,^9^, inactivated cell culture-derived HCV virions^10^, and virus-like particles (VLPs) presenting HCV envelope proteins^11^, have been examined for the ability to induce a humoral response against HCV. Although the resulting vaccines induced broadly neutralizing antibodies against the major genotypes of HCV, many problems still need to be addressed, including side effects, inconsistent efficacy among genotypes and strains, and the productivity and purity of the antigens. Recently, HCV E2 amino acid residues 412–423, a domain highly conserved among various genotypes, was shown to constitute a linear epitope that is recognized by broadly neutralizing monoclonal antibodies (mAbs) such as AP33 and HCV1^12^,^13^. However, antibodies recognizing this epitope are rare in natural infections, suggesting that this region is poorly immunogenic^14^,^15^.

Japanese encephalitis virus (JEV) is the leading cause of viral encephalitis with severe mortality in eastern and south-eastern Asia, and is estimated to be responsible for 67 900 cases annually^16^. JEV, a member of the genus Flavivirus within the family Flaviviridae, is an enveloped single-stranded positive-sense RNA virus with an 11-kb genome that is translated as a single large polyprotein. The polyprotein is co-translationally cleaved by host and viral proteases into three structural proteins–capsid, pre-membrane (prM), and envelope (E)–and seven non-structural proteins^17^. It is known that subviral particles (SVPs), which contain the lipid bilayer and the prM/M and E proteins but not nucleocapsid, are secreted from flavivirus-infected cells along with virions. Expression of the flavivirus prM and E glycoproteins in cells also induces formation and secretion of SVPs that share immunogenic properties with whole virions^18^,^19^,^20^,^21^. These observations suggest that SVPs could serve as vaccine candidates.

In the present study, we took advantage of the properties of JEV to develop a potential HCV vaccine. We established the efficient production of JEV SVPs harboring HCV-neutralizing epitopes for use as possible bivalent vaccine antigens. These particles efficiently elicited neutralizing antibodies against both JEV and HCV in immunized mice. Thus, a JEV particle-based platform displaying foreign epitopes on the particle surface could facilitate the development of a bivalent vaccine against JEV and other pathogens.

## Results

### Identification of the positions for foreign epitope insertion in JEV E protein

**Figure 1:**
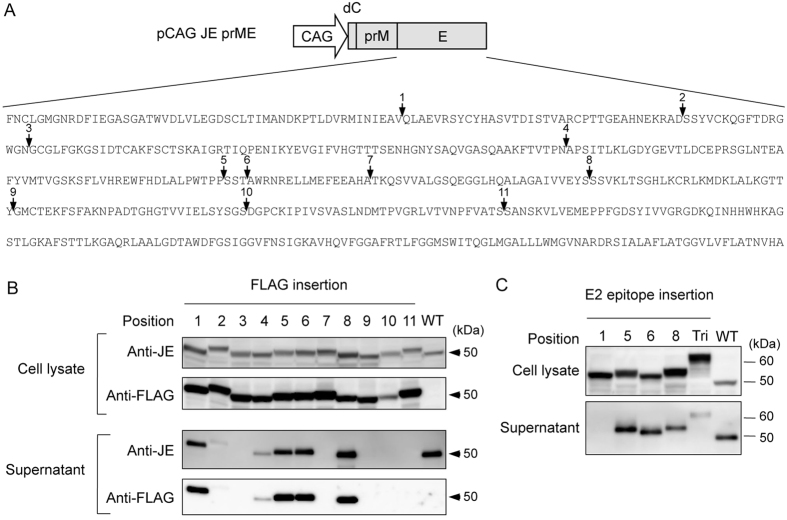
Identification of the positions for foreign epitope insertion in JEV E protein. (**A**) Schematic representation of plasmid used for expression of JEV prM and E. Amino acid sequence of the JEV E protein is shown. The positions for insertion of FLAG-tag or HCV E2 neutralization epitope are indicated by arrows. CAG: CAG promoter, dC: deleted partial C-terminal 22 aa of capsid. (**B**) Detection of JEV E protein in cell lysates and supernatants of 293T cells transfected with each plasmid. Detection was performed at 2 days post-transfection by immunoblotting using anti-JEV mouse serum or anti-FLAG antibody. (**C**) Detection of JEV E protein in cell lysates and supernatants of 293T cells transfected with each plasmid. Detection was performed at 2 days post-transfection by immunoblotting using anti-JEV mouse serum. For source data, see Supplementary Figure 1B,C.

We sought to identify positions within the ectodomain of the JEV envelope (E) protein that would tolerate foreign epitope insertion while allowing secretion of the modified E protein as a component of a particle. Using the published crystal structure of the E protein^22^, we selected 11 loop positions within the E protein that were not required contacts for dimerization of E (Fig. S1A). Complementary DNA sequences encoding the FLAG-tag were inserted into each of the selected positions of the E-encoding gene in the prME expression plasmid, as shown in Fig. 1A. These plasmids were used to transfect 293T cells, and lysates and supernatants of the resulting cells were screened by immunoblotting. As shown in Fig. 1B, all 11 constructs directed expression of FLAG-tag E protein in the cell lysate. Of the 11 constructs examined, 6 (position 1, 2, 4, 5, 6, 8) directed the expression of FLAG-tagged E protein that was detectable in the culture supernatant of the transfected cells; 4 of these insertion positions permitted the secretion of E protein to levels comparable to those seen with the construct lacking an insertion (WT). Therefore, we next constructed prME expression plasmids that encoded fusion proteins such that the linear neutralization epitope from HCV E2 (aa 412–423) was inserted into position 1, 5, 6, or 8 of the JEV E protein (the same positions that tolerated insertion of the FLAG-tag without impairment of secretion in the previous experiment). Of the 4 positions examined, positions 5, 6, and 8 permitted insertion of the HCV E2 neutralization epitope without impairment of secretion (Fig. 1C). Furthermore, a construct encoding JEV E protein containing HCV neutralization epitope inserted at all 3 sites (positions 5, 6, and 8; abbreviated as Tri in Fig. 1C) was also shown to direct the expression and secretion of JEV E protein.

### Display of HCV E2 neutralization epitope on the surface of SVPs

**Figure 2:**
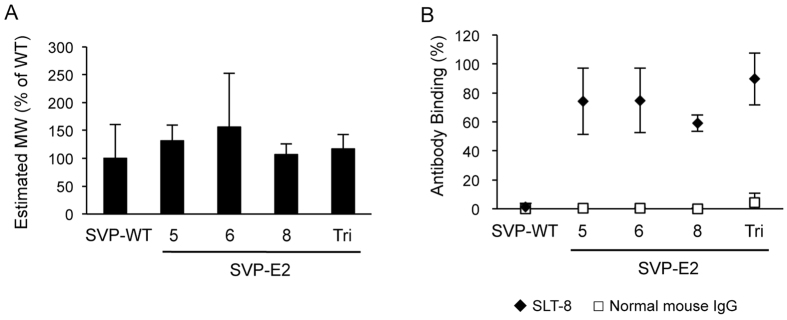
Characterization of JEV SVPs incorporating E protein with epitope insertions. (**A**) Estimated molecular masses of JEV SVPs with or without insertion were evaluated by FCS analysis. (**B**) The binding of SLT-8 monoclonal antibody or normal mouse IgG to JEV SVPs was analyzed by FCS analysis.

Flaviviruses prM and E proteins assemble and are secreted as SVPs with diameters of approximately 30 nm, even in the absence of other viral components^23^. To confirm that the secreted E protein containing the HCV E2 neutralization epitope was assembled into SVPs, the molecular masses of the secreted proteins in the supernatants of cells transfected with each prME expression plasmid were evaluated by fluorescence correlation spectroscopy (FCS). FCS measured fluorescence fluctuations of SVPs labeled by a fluorophore in a focused laser beam. From the time-dependent intensity fluctuations, diffusion time of the target particles was obtained by autocorrelation analyses (Fig. S2A), which gives information about molecular masses. As shown in Fig. 2A, particles generated by the cells expressing epitope-inserted JEV E (SVP-E2) had molecular masses that were almost equal to those of particles generated by cells expressing intact JEV E (without insertion; SVP-WT). We next examined whether the inserted epitopes were displayed on the surfaces of the particles. Each class of SVP was incubated with mAb SLT-8, which recognizes the linear neutralizing epitope from HCV E2 (aa 412–423), followed by incubation with fluorophore-labeled secondary antibody and FCS analysis. SLT-8 bound to each class of SVP-E2 particle in the supernatants, but not to the SVP-WT particles (Figs S2B and 2B). As a control, normal mouse IgG did not react with any of the SVPs (Figs S2C and 2B). These data suggested that the inserted HCV E2 neutralization epitope was displayed on the surface of the JEV SVPs.

### Immunization of mice with SVP-E2 elicits neutralizing antibodies against JEV and HCV

**Figure 3:**
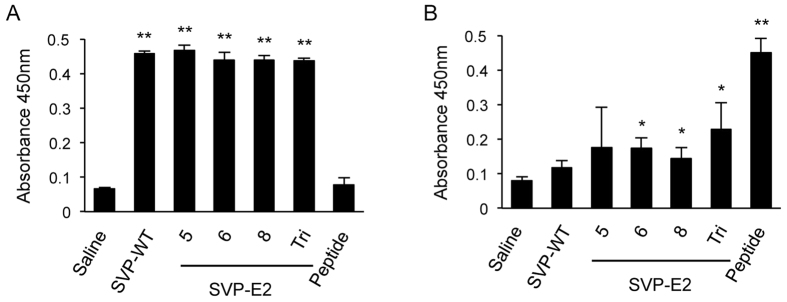
Detection of serum antibodies with specificity for JEV and HCV E2 peptide. Sera from immunized mice were diluted 1:200 and added to ELISA microtiter wells containing JEV particles at 35 ng/well (**A**) or HCV E2 (aa 404–423) peptide at 20 μM (**B**). Antibody binding was detected using HRP-conjugated anti-mouse secondary antibody. The statistical significance of differences between groups was evaluated using the Student’s *t*-test (*P < 0.01, **P < 0.001 vs. serum from saline-immunized control group).

In order to obtain antigen for immunization, we established stable 293T cell lines expressing each SVP. These cells were cultured in serum-free medium, and SVPs in the culture supernatant were concentrated by filtration over a 100-kDa cut-off membrane, followed by purification via size-exclusion chromatography (Fig. S3A,B). The presence of SVPs with diameters of 25–30 nm was confirmed by electron microscopy (Fig. S3C). Morphological differences between SVP-WT and SVP-E2 were not observed. To evaluate the immunogenicity of the SVPs, female BALB/c mice were intraperitoneally immunized 3 times using SVPs formulated with alum-CpG adjuvant. We analyzed the sera by ELISA to identify IgG antibodies directed against JEV particles and peptides corresponding to the inserted HCV E2-derived sequence. As shown in Fig. 3A, anti-JEV antibody was induced in all mice immunized with SVPs. In addition, sera obtained from mice immunized with SVP-E2/6, SVP-E2/8, or SVP-E2/Tri reacted with the HCV E2 peptide, although the titers as measured by ELISA absorbance signals of these sera were low compared to those of mice immunized with synthetic E2 peptides (aa 412–423), as shown in Fig. 3B. These results indicated that the SVP-E2 particles provided immunogenic presentation of the HCV neutralization epitope.

**Figure 4:**
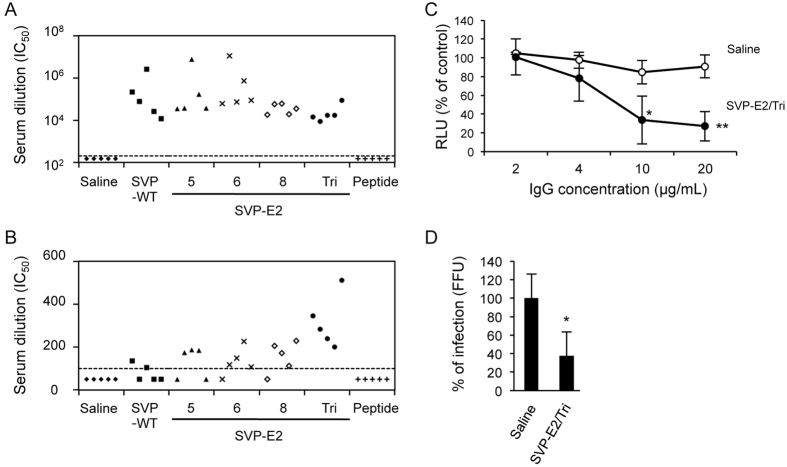
Neutralizing antibodies against JEV and HCV in sera from immunized mice. Sera were drawn from BALB/c mice at 1 week after third immunization. (**A**) JEV was preincubated with serially diluted sera for 1 h and then used to infect Vero cells. Cultures were incubated for 48 h and then infection was enumerated by counting E-positive foci by immunofluorescence. Values were calculated as the fold-dilution that resulted in 50% neutralization of JEV infection. The dotted line represents the lowest dilution tested (200-fold dilution), and values below this line are plotted at a value of 150 to represent less than 50% neutralization activity at lowest dilution tested. (**B**) HCVtcp derived from genotype-1b virus was preincubated with serially diluted sera for 1 h and then used to infect Huh7.5.1 cells. Cultures were incubated for 48 h and then luciferase activity of the cells was determined. Values are calculated as the fold-dilution that resulted in 50% neutralization of HCVtcp infection. The dotted line represents the lowest dilution tested (100-fold dilution), and values below this line are plotted at a value of 50 to represent less than 50% neutralization activity at lowest dilution tested. (**C**) HCVtcp derived from genotype-1b virus was preincubated for 1 h with serially diluted IgG purified from sera obtained from saline- or SVP-E2/Tri-immunized mice, and then used to infect Huh7.5.1 cells. Luciferase activity was determined at 48 h post-infection and is expressed relative to activity without IgG. (**D**) HCVcc derived from genotype-1b/2a chimeric viruses were preincubated for 1 h with serum (diluted 1:100) derived from saline- or SVP-E2/Tri-immunized mice, and then used to infect Huh7.5.1 cells. Cells were immunostained with anti-NS5A antibody at 5 days postinfection, and antigen-positive foci were counted and used to calculate infection titer. The statistical significance of differences between groups was evaluated using the Student’s *t*-test (*P < 0.01, **P < 0.001 vs. serum from saline-immunized control group).

We next examined whether the sera from immunized mice neutralized JEV and HCV infection. Sera from mice immunized with SVPs inhibited JEV infection *in vitro* (Fig. 4A). Furthermore, sera from mice immunized with SVP-E2/Tri, which contains HCV-neutralizing epitope inserted at 3 sites, showed the strongest neutralizing titer, although SVP-E2/5, 6, and 8 showed marginal effects (Fig. 4B). Purified IgG from mice immunized with SVP-E2/Tri also reduced infection by HCVtcp in a dose-dependent manner (Fig. 4C). In addition, sera from mice immunized with SVP-E2/Tri neutralized cell culture-produced HCV (HCVcc), as shown in Fig. 4D. These data suggested that SVP-E2/Tri elicited neutralizing antibodies against both JEV and HCV. It should be noted that sera from mice immunized with the synthetic peptide corresponding to the E2 neutralization epitope showed little neutralizing effect against HCV, although those sera did react to E2 peptide in an ELISA assay (Fig. 3B).

### Sera from mice immunized with SVP-E2/Tri neutralize infection by multi-genotypic HCV and by HCV that contains a mutation in the epitope region

**Figure 5:**
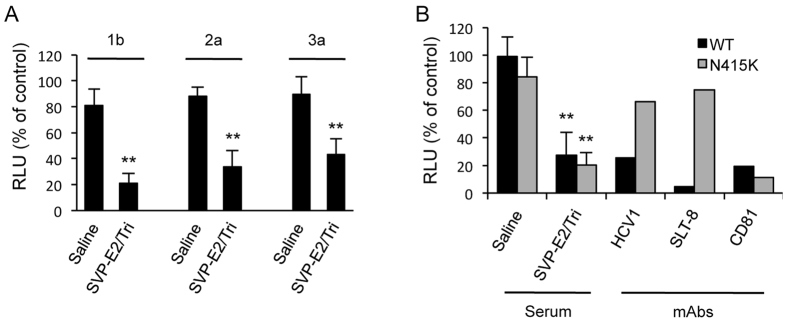
Neutralizing antibodies against multi-genotypic HCV and against HCV with a mutation in the epitope-encoding region. HCVtcp derived from genotype-1b, -2a, or -3a viruses (**A**) or HCVtcp with or without the N415K mutation (**B**) was preincubated for 1 h with monoclonal antibodies (HCV1, SLT8, or anti-CD81) at 1 μg/mL or with serum (diluted 1:100) derived from SVP-E2/Tri-immunized mice, and then used to infect Huh7.5.1 cells. Luciferase activity was determined at 48 h post-infection and is expressed relative to activity without antibodies. Data for serum are means of values obtained from five mice with error bars showing SD. The statistical significance of differences between groups was evaluated using the Student’s *t*-test (**P < 0.001 vs. serum from saline-immunized control group).

In order to evaluate the efficacy of SVP-E2/Tri as a vaccine for HCV, we also performed neutralization assays against multi-genotypic HCV. As shown in Fig. 5A, sera from mice immunized with SVP-E2/Tri reduced infection by genotype-2a and -3a HCVtcp, as seen above for genotype-1b HCVtcp. These data suggested that SVP-E2/Tri elicited broadly neutralizing antibodies against HCV.

Antibodies (e.g., AP33 or HCV1) with specificity for aa 412–423 of HCV E2 have been shown to neutralize pangenotypic HCV infection^12^,^13^. However, single missense mutations (e.g., encoding N415K) in the epitope region have been shown to render HCV resistant to these antibodies^24^,^25^. Therefore, we examined whether the sera from mice immunized with SVP-E2/Tri neutralized infection by HCV harboring the N415K mutation. While HCVtcp lacking a mutation in the epitope region (WT) was highly susceptible to neutralization by both HCV1 and SLT-8 antibodies, HCVtcp with the N415K mutation was resistant to these mAbs (Fig. 5B). In contrast, sera from mice immunized with SVP-E2/Tri inhibited infection by HCVtcp with the N415K mutation. These data suggested that sera from mice immunized with SVP-E2/Tri are still able to neutralize HCV containing at least one defined escape mutation, in contrast to the resistance of mutated HCV to mAbs such as AP33 or HCV1.

### SVPs with E1 epitope insertion also elicit neutralizing antibodies against JEV and HCV

**Figure 6:**
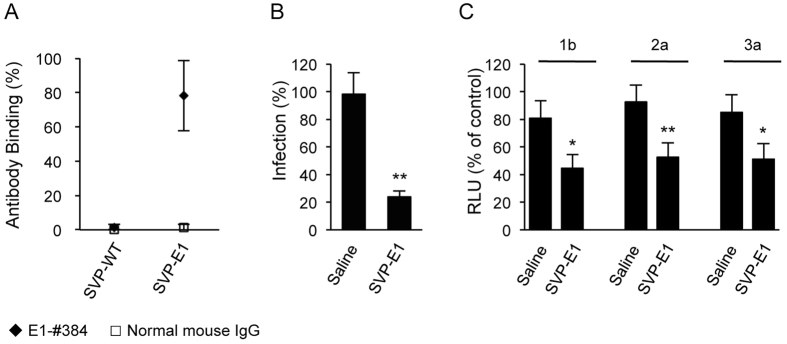
Neutralizing antibodies against JEV and HCV in sera of mice immunized with SVP-E1. (**A**) The binding of monoclonal antibody E1-#384 or normal mouse IgG to JEV SVPs was determined by FCS analysis. (**B**) JEV was pre-incubated with 5000-fold diluted sera for 1 h and then used to infect Vero cells. Cultures were incubated for 48 h and infection then was enumerated by counting E-positive foci using immunofluorescence. Infectivity is expressed as the percentage of virus infection without serum. (**C**) HCVtcp derived from genotype-1b, -2a, or -3a viruses were preincubated for 1 h with 100-fold diluted sera from mice immunized with saline or SVP-E1, and then used to infect Huh7.5.1 cells. Luciferase activity was determined at 48 h post-infection and is expressed relative to activity without serum. The statistical significance of differences between groups was evaluated using the Student’s *t*-test (*P < 0.01, **P < 0.001 vs. serum from saline-immunized control group).

Although E2 is the primary target for induction of neutralizing antibodies against HCV, the other envelope protein (E1) also has been reported to be a target for neutralizing antibodies^26^,^27^. We confirmed those reports by demonstrating that the anti-E1 mouse mAb E1-#384 neutralized multi-genotypic HCV (Fig. S4A). To define the epitopes recognized by the E1-#384 antibody, a panel of overlapping peptides derived from HCV E1 was synthesized, and antibody reactivity to the peptides was analyzed by an indirect immunoassay. The minimum epitope of E1-#384 was defined as aa 226–238 of E1 (Fig. S4B,C). We therefore generated and isolated SVPs (designated SVP-E1) from a cell line expressing a fusion protein consisting of the E1 neutralizing epitope inserted at position 5 of JEV E, and confirmed that the E1 epitope was displayed on the surface of these particles (Fig. 6A). As seen above with SVP-E2, sera from mice immunized with SVP-E1 inhibited infection by JEV and HCV (Fig. 6B,C). The combination of our SVP-E1 and SVP-E2 results suggested that a JEV particle-based platform can be used for displaying foreign epitopes on viral particle surfaces.

## Discussion

Vaccination is the most cost-effective strategy to control many infectious diseases. Among vaccine antigens, VLP-based vaccines are considered highly immunogenic, eliciting high-titer neutralizing antibody responses more efficiently than do individual proteins or peptides. This superior performance reflects the effective recognition of VLP vaccines by B cells, with resulting stimulation of B cell signaling to facilitate the generation of high-titer, specific antibodies^28^.

In the present study, we explored positions in the JEV envelope protein that are both exposed on the surface of the viral particle and tolerant of foreign epitope insertion without impairment of secretion. Among the 11 positions we examined in the JEV E protein, 6 positions tolerated insertion of the FLAG-tag epitope, and 3 of those positions permitted insertion of the 12-aa HCV E2 neutralizing epitope without impairment of secretion. For the tolerant positions, the inserted HCV-neutralizing epitope was shown to be displayed on the surface of the resulting SVPs. Furthermore, particles could incorporate JEV E protein with foreign epitope inserted at all 3 positions at the same time. To our knowledge, this is the first demonstration that flavivirus SVPs can be used to display inserted foreign epitope on the surface of a particle.

No prophylactic vaccine for HCV infection currently exists, although several HCV vaccines are in development^29^. A domain (aa 412–423) of HCV E2 that is involved in CD81 binding has been shown to constitute a conserved linear epitope that is targeted by broadly neutralizing antibodies such as AP33 or HCV1^12^,^13^. Notably, HCV1 has been used to prevent, as well as to treat, HCV infection in a chimpanzee model, although large amounts of antibody are needed^25^. However, these antibodies are unable to neutralize viruses containing single escape mutations in the epitope-encoding region^24^,^25^. Furthermore, weak immune responses against this epitope were reported in infected patients^14^,^15^. To overcome these problems, efficient elicitation of polyclonal antibodies against aa 412 to 423 of HCV E2 was attempted.

Recently, the structure of the HCV E2 core domain was resolved^30^,^31^, although the N-terminal region containing HVR1 and the conserved linear epitope remained disordered in this structure. However, crystal structure of the Fab fragment of AP33 or HCV1 in complex with the epitope peptide (aa 412 to 423) revealed that the peptide forms an antiparallel β-hairpin loop^32^,^33^. In addition, crystal structure of the same epitope with the Fab fragment of the 3/11 antibody (which displays a neutralizing activity similar to that of AP33 and HCV1) revealed that the 3/11 antibody recognized a different conformation of the same epitope^34^. These data suggest that the conformation of the aa-412-to-423 region of HCV E2 is flexible. In the present study, the sera of mice immunized with synthetic E2 peptide reacted strongly with synthetic E2 peptide, although the same sera did not demonstrate detectable neutralizing activity against genotype-1b HCVtcp, as shown in Figs 3B and 4B. In contrast, immunization of mice with SVP-E2/Tri elicited neutralizing antibodies against HCV. These results suggested that binding of antibody to the peptide is not sufficient for neutralization. Although the conformation of the E2 epitope appears to be flexible, we speculate that the specific structure of the peptide as displayed on the surface of JEV-SVP is more efficient at eliciting antibodies with neutralizing activity against HCV.

Given that the aa-412-to-423 region of E2 is conserved among different genotypes, it is not surprising that SVP-E2/Tri elicited broadly neutralizing antibodies effective against various HCV genotypes (Fig. 5A). Within the aa-412-to-423 region of E2, isoleucine at 414 is observed less frequently in viruses of genotypes 3a, 3b, and 4 as judged by searches of the Hepatitis Virus Database (http://s2as02.genes.nig.ac.jp/), as shown in Table 1. Given that sera from mice immunized with SVP-E2/Tri were effective in reducing infection by genotype-3a HCVtcp (Fig. 5A), which encodes valine at amino acid position 414, anti-sera used in this study might be binding most of the viral antigens from the patient serum. In addition, these sera inhibited infection by HCVtcp harboring the N415K mutation, a lesion that renders HCV resistant to mAb HCV1 or SLT-8 (antibodies with specificity for this region of HCV E2). We presume that SVP-E2/Tri induces polyclonal antibodies against the E2 epitope, such that some of the antibodies are able to recognize sub-epitopes not affected by the single escape mutation, even though that position is essential for recognition by mAbs such as HCV1.

Although E2 is the primary target for induction of neutralizing antibodies against HCV, neutralizing antibodies against E1 also have been reported^26^,^27^,^35^. We found that anti-E1 mouse mAb E1-#384 decreased multi-genotypic HCV infection. Epitope-mapping analysis of the antibody revealed that the epitope recognized by mAb E1-#384 does not correspond to the same sequence as that of a previously reported neutralizing epitope of E1^27^. Therefore, we tested this novel E1 neutralizing epitope using our JEV particle-based platform. The sequence of this E1 region is not well conserved among different HCV genotypes (Fig. S4D); nonetheless, sera of mice immunized with our SVP-E1 particles incorporating the E1 epitope from HCV genotype 1b neutralized HCVtcp derived from viruses of genotype 1b, 2a, and 3a. These data indicated that the E1 epitope also would be an appropriate target for induction of neutralizing antibodies against HCV.

Flavivirus SVPs share immunogenic properties with whole virions, so that SVPs can serve as immunogens for eliciting neutralizing antibodies^18^,^19^. In the present work, we showed that JEV SVPs incorporating E protein with insertions at up to 3 positions still elicited neutralizing antibodies against JEV. This result demonstrated that the antigenicity of the JEV E protein as required for induction of neutralizing antibodies is not impaired by insertion of foreign peptides at selected positions. Since flavivirus SVPs are non-infectious and are efficiently secreted from prM- and E-expressing cells, use of the JEV particle-based platform for production and purification of antigens is expected to be beneficial in terms of cost and safety. Further study will be needed to optimize various parameters such as adjuvant, dose, schedule, and mode of delivery, especially for application in a primate model.

The work described here indicates that vaccines derived from JEV SVPs incorporating E protein with HCV epitope insertion(s) are capable of eliciting broadly cross-neutralizing antibodies against both HCV and JEV. A JEV particle-based platform displaying foreign epitopes on the surface of the particle could facilitate the development of a bivalent vaccine against JEV and other pathogens.

## Materials & Methods

### Cell cultures

Human embryonic kidney 293T cells, human hepatoma-derived Huh7.5.1 cells, Vero cells, and GP2-293 cells for retroviral packaging (Clontech) were maintained in Dulbecco’s Modified Eagle’s medium (DMEM) supplemented with nonessential amino acids, 100 U of penicillin/mL, 100 μg of streptomycin/mL, and 10% fetal bovine serum. Cultures were grown at 37 °C in a 5% CO_2_ incubator.

### Plasmid

Plasmids pCAG-JEprME for expression of JEV prM/E was previously described^36^. For the construction of the insertion-containing prM/E-encoding plasmids, cDNAs encoding FLAG-tag (DYKDDDDK), E2 neutralization epitope (aa 412–423; QLINTNGSWHIN), or E1 neutralization epitope (aa 226–238; CVPCVRENNSSRC) were inserted into pCAG-JEprME.

In order to construct a retrovirus vector for dicistronic expression of GFP and JEV prME proteins, the neomycin-resistance gene of pQCXIN (Clontech) was replaced with the GFP-encoding gene from pGreen Lantern (Life Technologies), and the prM-E-encoding gene (with or without foreign epitope) was inserted downstream of (and under the control of) the CMV promoter. For the production of HCVtcp, pHH/SGR-Gluc/NS3m was constructed by replacement of the Gluc fragment of pCMV-GLuc (NEB) with a firefly luciferase-encoding fragment of pHH/SGR-Luc harboring an NS3 mutation^37^. For generation of HCVtcp from genotype-3a virus, a C-NS2 expression plasmid derived from S310^38^ was constructed by using PCR and confirmed by sequencing. For generation of genotype-1b/2a chimeric HCVcc, the cDNA coding for the first transmembrane region of NS2 (33 amino acids) in pHHJFH1 was replaced with the corresponding sequence from the THpa isolate^37^. The plasmid sequences used in this study are available from the authors upon request.

### DNA transfection

Monolayers of 293T cells were transfected with plasmid DNA using FuGENE 6 transfection reagent (Promega) in accordance with the manufacturer’s instructions.

### Establishment of stable cell lines producing JEV SVPs

GP2-293 cells were co-transfected with pVSV-G and each of the constructed retrovirus plasmids using PEI reagent. At 48 h post-transfection, supernatants of cells containing retroviruses were collected. 293T cells were infected with the resulting retroviruses. The cells expressing high levels of GFP were sorted by flow cytometry.

### Purification of SVPs

293T cells stably producing SVPs were incubated in Opti-MEM (Invitrogen). Supernatant was filtered through a 0.45-μm filter to remove cell debris, then concentrated by ultrafiltration using an Amicon Ultra 100K filter (Merck Millipore). Concentrated supernatant was fractionated by chromatography using Superdex 200 (GE). Fractions containing SVPs were pooled for use in immunization.

### Antibodies

Mouse mAbs against the FLAG-tag (M2) were obtained from Sigma-Aldrich. The mouse mAb against flavivirus group antigen (D1-4G2-4-15) and Normal mouse IgG were obtained from Merck Millipore. Anti-JEV mouse serum was developed by infection of mouse with JEV. The mouse mAb SLT-8, which recognizes amino acids 412–423 of the HCV E2 glycoprotein, was developed by immunization of the mouse with E2 envelope glycoprotein derived from the HCV TH clone (genotype 1b) (Y. Shibuya, N. Nakamura, and T. Wakita, unpublished data). The mouse mAb against HCV E1 (E1-#384) was developed by immunization of mouse with recombinant HCV E1 derived from a genotype-1b virus. Anti-NS5A antibodies were rabbit polyclonal antibodies raised against synthetic peptides, as described elsewhere^37^.

### FCS analysis

For evaluation of estimated molecular masses of the particles, the supernatants of cells transfected with prME-expressing plasmids were reacted with Alexa Fluor 488-NHS (Invitrogen); free dye subsequently was removed using a gel filtration column. For analysis of epitope on the surfaces of the particles, the supernatants were incubated with SLT-8 antibody or normal mouse IgG for 30 min at room temperature, followed by incubation with Alexa 488-labeled goat anti-mouse IgG (Invitrogen) for 30 min at room temperature. A commercial FCS setup (FCS-101B, Hamamatsu Photonics, Shizuoka, Japan), which is equipped with a water-immersion objective (UPAPLO 40 × /1.2 W, Olympus), was used. The 473-nm line of a semiconductor laser (output power 1 mW) was applied and attenuated adequately with light filters. The laser beam was focused at about 0.2 mm above the bottom of cover glasses in a typical sample droplet volume of 20 μL. Fluorescence intensity data through an optical filter (>500 nm) located adjacent to the photo multiplier tube were collected. Autocorrelation analysis of the fluorescence intensity fluctuations was performed using the control software supplied by the manufacturer.

### Generation of viruses

HCVtcp was generated by transfection of Huh7.5.1 cells with pHH/SGR-Gluc/NS3m and C-NS2 plasmid derived from THpa (genotype 1b) or JFH1 (genotype 2a), as described previously^37^. For generation of HCVtcp from genotype-3a virus, C-NS2 expression plasmid derived from S310^38^ was used. For generation of HCVtcp with the N415K mutation, the mutation was introduced (via PCR) into the C-NS2 expression plasmid derived from THpa. HCVcc was generated as described previously^37^. The JEV Nakayama strain was described previously^36^.

### Immunization of mice

Specified pathogen-free female BALB/c mice (4 weeks old) were purchased from SLC Japan. Aliquots (20 μg) of SVP-WT, SVP-E1, SVP-E2, or KLH-conjugated E2 (aa 412–423) peptides were formulated with alum (200 μg, Thermo) and CpG (25 μg, Sigma) for immunization. Each formulation (0.1-mL volume) was injected intraperitoneally (into respective animals) at weeks 1, 3, and 5, and blood was collected at weeks 0, 2, 4, and 6. Control animals were injected according to the same regimen with an equivalent volume of saline, and blood was collected according to the same schedule. Blood samples were collected in a Bloodsepar (Takara) and centrifuged at 2500× g for 2 min at room temperature. The supernatants were collected as sera and were heat-inactivated at 56 °C for 30 min for use in ELISA and neutralization assays. All animal experiments were approved by the Animal Care and Use Committee of National Institute of Infectious Diseases and were carried out in accordance with the approved guidelines. Mouse IgG was purified using PureSpeed ProG resin (Rainin) in accordance with the manufacturer’s instructions.

### Enzyme-linked immunosorbent assay

HCV E2 (aa 404–423) peptide (20 μM) or JEV particles (35 μg in TBS (50 mM Tris-HCl, 150 mM NaCl)) were adsorbed onto Maxisorp 96-well plates (Nunc) by overnight incubation at 4 °C. The wells were washed, blocked with Block Ace (Snow Brand Milk Products Co.), and then washed with phosphate-buffered saline containing 0.05% Tween20 (TPBS). Sera (1/200 dilution) were added to each well, and plates were incubated for 1.5 hours at room temperature. The sera then were discarded and each well was washed thrice with TPBS. HRP-coupled anti-mouse IgG (1/5,000 dilution; GE Healthcare) was added to each well and plates were incubated at room temperature for 1 hour prior to addition of substrate (peroxidase detection kit; Sumitomo Bakelite). The absorbance at 450 nm was determined with an ELISA reader (BioRad).

### Neutralization assay

For HCV neutralization assay, HCVtcp was incubated with serial dilutions of sera or of purified immunoglobulin at room temperature for 1 h. Huh7.5.1 cells were infected with the resulting mixtures for 5 hours at 37 °C. Following infection, supernatant was removed and cells were incubated with fresh culture medium. Luciferase activity was determined at 2 days post-infection using the BioLux Gaussia Luciferase Assay Kit (NEB). All luciferase assays were performed in triplicate. Titration of HCVcc was described previously^37^.

For JEV neutralization assay, the JEV Nakayama strain was incubated with serial dilutions of sera at room temperature for 1 h. Vero cells were infected with the resulting mixtures for 5 hours at 37 °C, and the supernatants then were replaced with fresh medium containing 10% FBS and 0.8% carboxymethyl cellulose. Following incubation for 48 hours at 37 °C, the monolayers were fixed and immunostained with the anti-E antibody (D1-4G2-4-15, Millipore) followed by an Alexa Fluor 488-conjugated anti-mouse secondary antibody (Invitrogen). Stained foci were counted and used to calculate the titers of focus-forming units (FFU)/mL.

## Additional Information

**How to cite this article**: Saga, R. *et al*. Bivalent vaccine platform based on Japanese encephalitis virus (JEV) elicits neutralizing antibodies against JEV and hepatitis C virus. *Sci. Rep.* **6**, 28688; doi: 10.1038/srep28688 (2016).

## Supplementary Material

Supplementary Information

## Figures and Tables

**Table 1:** Frequency of amino acid residues 412–423 of the epitope used in the study in the sequence database of HCV^a^.

Genotype	Tested	412	413	414	415	416	417	418	419	420	421	422	423
		Q	L	I	N	T	N	G	S	W	H	I	N
		% of strains											
1a	483	93.6	100	78.5	97.3	82.4	95.9	99.6	99.6	100	99.8	97.7	100
1b	364	99.2	100	56.0	98.4	92.9	99.2	100	99.7	100	100	98.4	100
2a	32	100.0	100	93.8	90.6	84.4	93.8	100	100	100	100	93.8	100
2b	90	48.9	100	88.9	90.0	78.9	100	100	98.9	100	100	98.9	100
3a	34	100.0	100	11.8	94.1	100	100	100	100	100	100	100	100
3b	5	80.0	100	0	100	40.0	100	100	100	100	100	100	100
4	70	82.9	100	25.7	91.4	68.6	98.6	100	100	100	100	88.6	100
5	9	100.0	88.9	77.8	88.9	88.9	100	100	100	100	100	100	100
6	126	93.7	100	71.4	95.2	73.0	97.6	99.2	100	100	100	83.3	100

^a^http://s2as02.genes.nig.ac.jp/.
