# Supplementary material for: Bivalent vaccine platform based on Japanese encephalitis virus (JEV) elicits neutralizing antibodies against JEV and hepatitis C virus
Source: Sci Rep. 2016 Jun 27;6:28688. doi: 10.1038/srep28688 (PMC4922013; doi:10.1038/srep28688)
Supplement: Supplementary Information [file srep28688-s1.pdf]

Bivalent vaccine platform based on Japanese encephalitis virus (JEV) elicits neutralizing antibodies against JEV and hepatitis C virus

Ryohei Saga[1 2], Akira Fujimoto[1], Noriyuki Watanabe[1], Mami Matsuda[1], Makoto Hasegawa[3], Koichi Watashi[1], Hideki Aizaki[1], Noriko Nakamura[4], Shigeru Tajima[5], Tomohiko Takasaki[5], Eiji Konishi[6, 7], Takanobu Kato[1], Michinori Kohara[8], Haruko Takeyama[2], Takaji Wakita[1], Ryosuke Suzuki[1]

[1]Department of Virology II, National Institute of Infectious Diseases, Japan

[2]Department of Life Science and Medical Bioscience, Waseda University, Japan

[3]Department of Bioscience, Faculty of Bioscience, Nagahama Institute of Bio-Science and Technology, Japan

[4]Pharmaceutical Research Laboratories, Toray Industries, Inc., Japan

[5]Department of Virology I, National Institute of Infectious Diseases, Japan

[6]BIKEN Endowed Department of Dengue Vaccine Development Faculty of Tropical Medicine, Mahidol University, Thailand

[7]BIKEN Endowed Department of Dengue Vaccine Development, Research Institute for Microbial Diseases, Osaka University, Japan

[8]Department of Microbiology and Cell Biology, Tokyo Metropolitan Institute of Medical Science, Japan

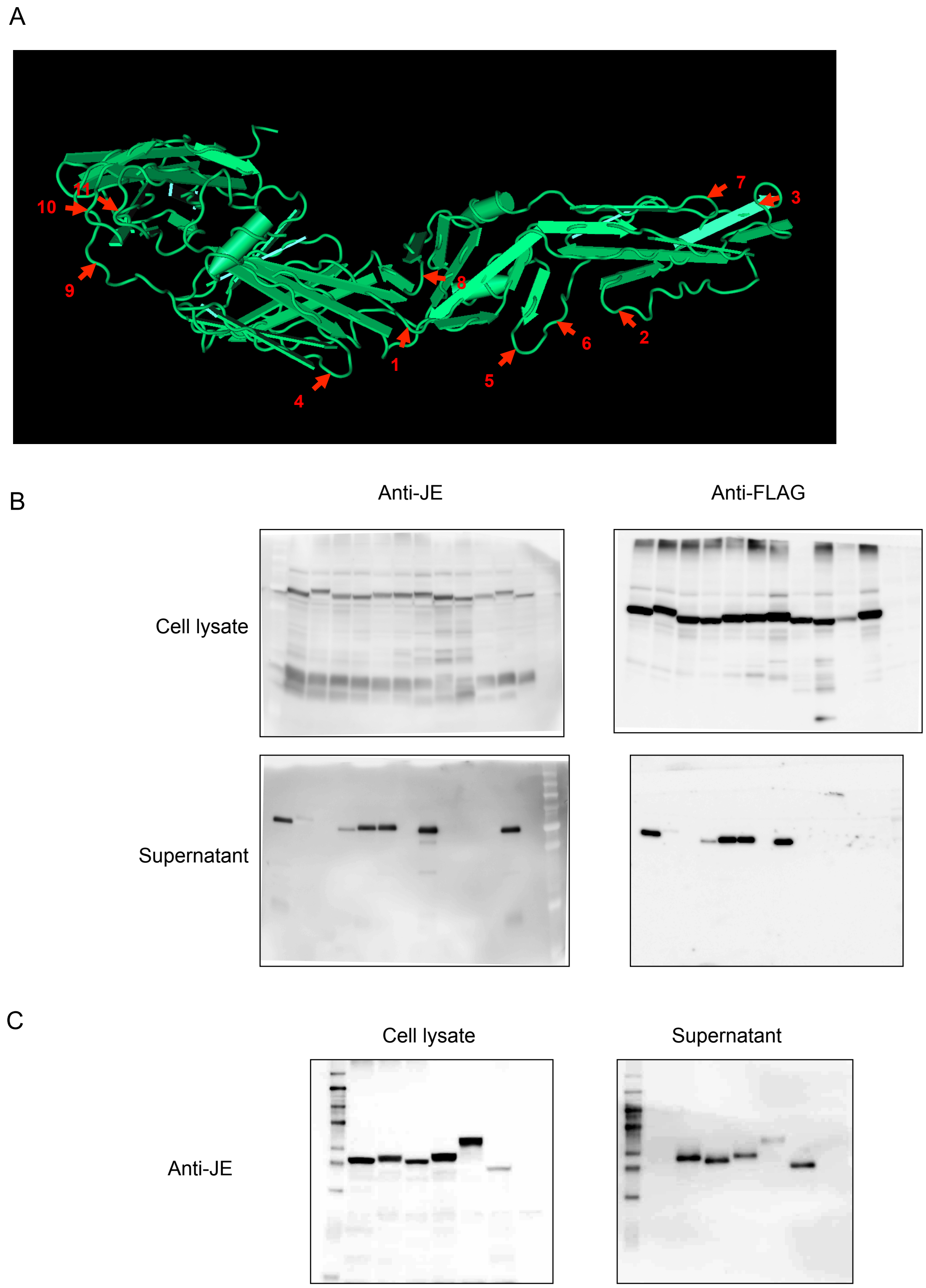

Supplementary Figure 1. Identification of the positions for foreign epitope insertion in JEV E protein. (A) Crystal structure (top view) of the JEV E ectodomain {Luca, V. C. *et al.* Crystal Structure of the Japanese Encephalitis Virus Envelope Protein. *Journal of virology* **86**, 2337-2346, DOI:10.1128/JVI.06072-11 (2011)}. The positions corresponding to site of epitope insertion are indicated by arrows. (B) Source data of Fig. 1B. (C) Source data of Fig. 1C.

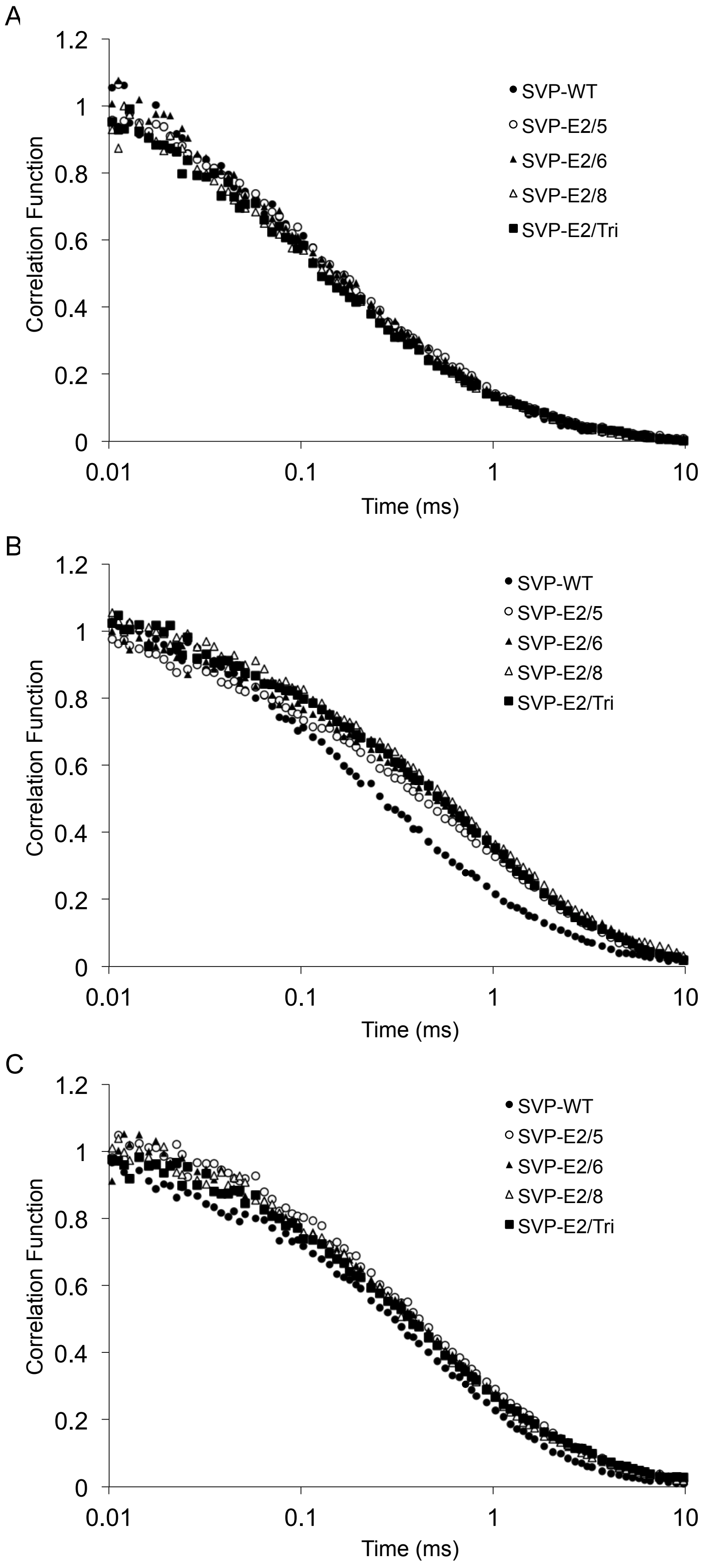

Supplementary Figure 2. FCS analysis of each class of SVP. (A) FCS autocorrelation curves of the fluorophore-labeled SVPs were generated from fluorescence fluctuations. Correlation function was used to determine the average diffusion time of the particles inside the FCS focus during measurement time. (B and C) FCS autocorrelation curves of the each class of SVP incubated with SLT-8 (B) or normal mouse IgG (C), followed by incubation with fluorophore-labeled anti-mouse IgG. Correlation function was used to determine the binding rate of the antibodies.

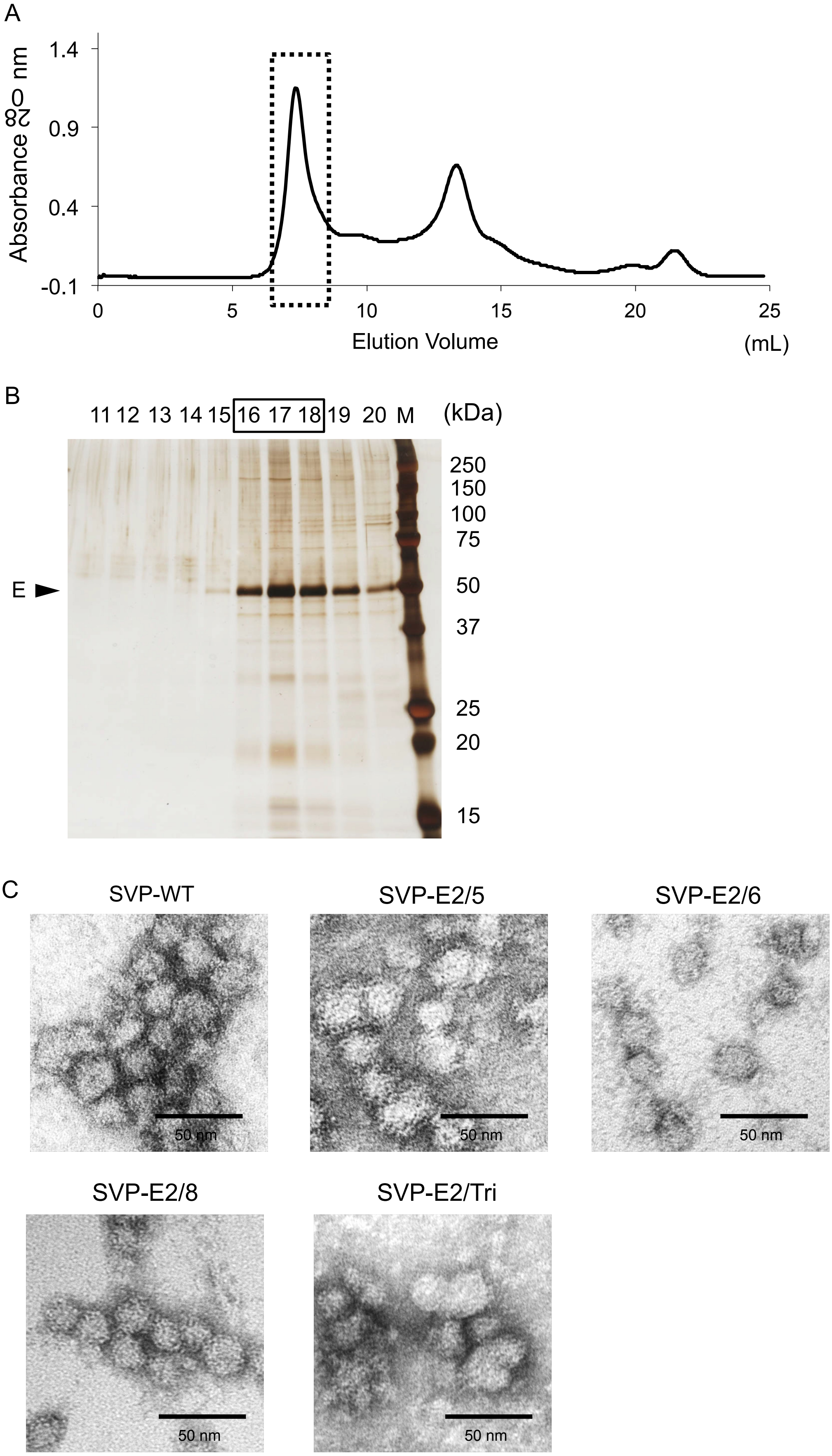

Supplementary Figure 3. Purification of JEV SVPs. (A) Representative gel-filtration chromatogram of JEV SVPs from culture supernatant. The dotted box indicates the peak of SVP. (B) Fractions (0.5 mL each) were collected and analyzed by SDS-PAGE with protein staining. Fractions #16-18 were pooled and used for immunization. (C) Electron microscopy of JEV SVPs. Purified SVPs were negatively stained with uranyl acetate and examined with a Hitachi H7100 microscope. Scale bars represent 50 nm.

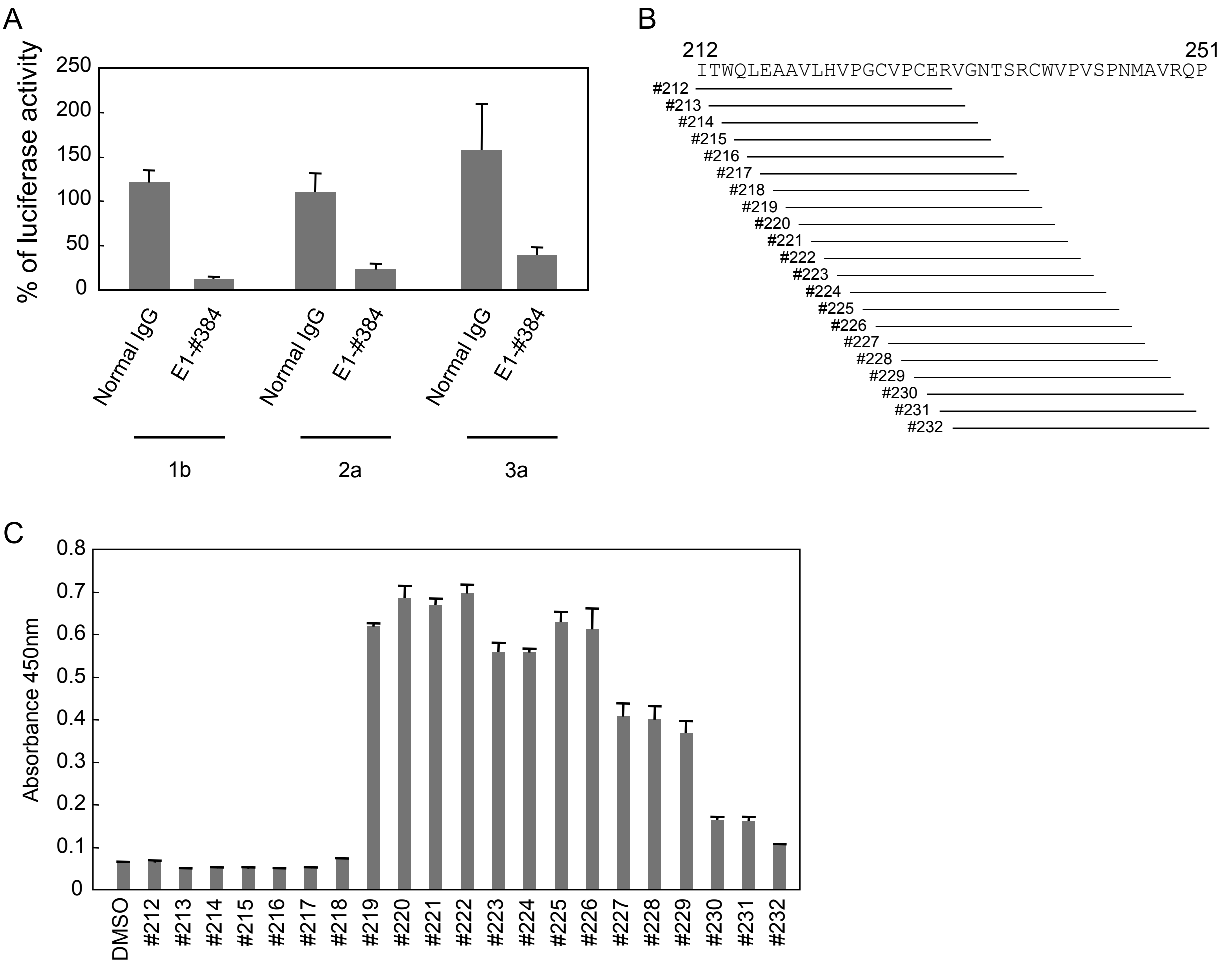

D

Inserted epitope		CVPCVRENNSSRC
(genotype)	(strain)	
1b	THpa	*************
2a	JFH-1	****E*VG*T***
3a	S310	*I***QDG*K*T*

Supplementary Figure 4. Characterization of anti-E1 monoclonal antibody E1-#384. (A) HCVtcp derived from genotype-1b, -2a, or -3a viruses was preincubated with E1-#384 at 10 μg/mL for 1 h and then used to infect Huh7.5.1 cells. Luciferase activity was determined at 72 h post-infection and is expressed relative to activity without antibodies. (B) Schematic figure of 20-mer HCV E1 overlapping peptides corresponding to aa 212-251 of E1 as encoded by the JFH1 strain. Each black line represents an overlapping peptide and its corresponding location. (C) Epitope mapping of E1-#384 antibody. Aliquots of 0.4 mg/mL antibody were added to ELISA microtiter wells containing each peptide at 20 μM. Antibody binding was detected with HRP-conjugated anti-mouse secondary antibody. (D) Alignment of amino acid sequences of the inserted E1 epitope among various HCV genotypes.
